# Editorial: Multidisciplinary perspectives on team sports: contextualizing training and competition demands, volume II

**DOI:** 10.3389/fpsyg.2026.1891759

**Published:** 2026-06-25

**Authors:** Pierpaolo Sansone, Anthony S. Leicht, Vincenzo Rago, Miguel Angel Gomez-Ruano

**Affiliations:** 1 Department of Education and Sport Sciences, Pegaso Telematic University, Naples, Italy; 2 Sport and Exercise Sciences, James Cook University, Townsville, QLD, Australia; 3 High Performance Sports Institute, SpexSG, Singapore, Singapore; 4 Facultad de Ciencias de la Actividad Física y el Deporte, Universidad Politécnica de Madrid, Madrid, Spain

**Keywords:** basketball, coaching, complex systems, soccer, sport physiology, sport psychology

After the first edition of the Research Topic (Sansone et al.), the second one collected an impressive number of 23 studies, spanning from physiological demands and training design to mental fatigue, psychological wellbeing, coaching behavior, and in-competition decision-making. Altogether, the obtained evidence provides valuable and novel information on team sport training and performance, offering a multidimensional framework that accounts for the cognitive, emotional, and social dimensions of sport alongside the physical and physiological aspects.

## Physical performance in training and competition contexts

Several studies examined how training structure shaped physical and physiological responses. A review of small-sided games (SSGs) in soccer demonstrated that numerical imbalances produce marked differences in physiological load, technical output, and tactical behavior (Rumpf et al.). Teams in numerical superiority recorded lower heart rates and less high-intensity running, yet improved passing and spatial coverage, providing coaches with a powerful lever for differentiating training stimuli within a single session. In futsal, a complementary investigation classified six distinct training task types and quantified their physiological and biomechanical demands across three professional seasons (Serrano et al.). Superiority/inferiority tasks and full-court exercises produced the highest loads, while introductory and analytical tasks functioned as effective recovery stimuli. Visualizing task demands, through a four-quadrant effort model, offered practitioners an accessible planning tool. In youth basketball, a constraint-based approach yielded further insight with an eight-week, dribbling-restriction, intervention producing superior aerobic adaptations compared to free-play SSGs (Li et al.), suggesting that even simple rule modifications can direct physiological development without altering the sport-specific context. Another basketball study found that verbal coach encouragement during youth SSGs produced significantly higher heart rates, perceived exertion, enjoyment, and technical performance compared to sessions without encouragement (Yilmaz et al.). Effect sizes were large, underscoring the potency of simple motivational behaviors in shaping both psychophysiological states and skill execution. Examining female volleyball, a cross-sectional study confirmed that countermovement jump and single-leg hop tests predicted sprint and agility performance, reinforcing the value of neuromuscular screening during routine monitoring (Akgün et al.). Next, a head-to-head comparison of elite and amateur cyclists (Cesanelli et al.) identified that amateurs, who exhibited significantly lower maximal aerobic power, completed the five-stage event with key deficits physiologically but also for race tactics and effort distribution.

Two studies addressed practical monitoring and performance-enhancing techniques. An accelerometer-based study of 35 professional rugby league players across a full competitive season reported that ~2/3 of match play involved high-intensity activity with limited positional differences in load distribution (Turner et al.). Finally, acute caffeine supplementation was shown to partially restore aerobic capacity, measured via the 30–15 Intermittent Fitness Test, though it did not fully reverse deficits in anaerobic power for sleep-restricted male, collegiate, soccer players (Cheng et al.). This finding adds nuance to caffeine's utility as a recovery strategy, indicating that its benefits for sleep restriction are domain-specific.

## Mental fatigue in team sports athletes

Mental fatigue has attracted substantial research attention, and the current Research Topic advances the field by scrutinizing the conditions under which it genuinely impairs performance. Acute mental fatigue negatively affected sprint and reaction times in university-level cricketers, regardless of whether it was induced by a cognitive Stroop task or a sport-specific video stream (Evans et al.). This finding was notable because it implicated passive media consumption as a potential performance hazard in pre-competition routines. On the other hand, in semiprofessional male basketball, subjective ratings of mental fatigue collected the day before a game showed no association with any game-related statistics across 17 official matches (Sansone et al.). This result was scientifically important as it cautioned against over-interpreting perceived mental fatigue, and highlighted the gap between laboratory protocols and the complex, high-motivation context of real competition. Supporting this finding, a study of professional female basketball players reported that cognitive load, assessed via perceived cognitive exertion and heart rate variability, followed a predictable weekly pattern, peaking mid-microcycle, with meaningful relationships to both external and internal load (Fuster et al.). These findings suggested that monitoring all three load components together provided a richer picture of athlete status. Lastly, a prospective cohort study of adolescent soccer players reproted that academic stress and mental fatigue predicted higher ratings of perceived exertion during training, but did not affect physiological load as measured by TRIMP (Nijland et al.). These authors recommended that coaches of dual-career athletes should treat elevated perceived effort with particular sensitivity, and recognize that it may signal scholastic pressure rather than true physical overload.

## Contextual factors and perceptual-motor skills

A meta-analytic synthesis of 22 studies examining visual and perceptual-cognitive skills in team sport athletes revealed a clear hierarchy of associations (Yang et al.). Multiple object tracking showed the strongest link with sport-specific performance, followed by visual attention and visual search (both moderate linkages). Choice and simple reaction times exhibted moderate negative correlations with performance, while basic perceptual skills such as depth perception were essentially unrelated to performance. These findings affirmed that the cognitive dimensions of vision, rather than raw sensory acuity, were relevant targets for assessment and training in team sports. Two studies probed the strategic use of timeouts during 1,200 elite basketball games with interesting findings. Analyzing over 4,000 timeouts from more than 1,200 elite games, researchers found that timeouts significantly increasing team momentum, but only when a team was losing, whereascalling a timeout when winning tended to reduce momentum (Qiu et al.). In water polo, an analysis of European Championship matches identified that power-plays were more successful without a preceding timeout than with one, at both senior and youth levels (Lupo et al.). Taken together, these findings challenged common coaching conventions and argued for evidence-based refinement of in-game tactical decisions. Finally, home advantage, long assumed to derive partly from crowd support, received empirical scrutiny using a natural experiment from the 2021 K League 1 season, where regional COVID-19 policies created unplanned variation in spectator presence (Lee et al.). Crowds improved tactical performance for home teams, with more shots on target, but the effects on away teams were more pronounced with lower winning rates and more goals conceded. This asymmetric finding suggested that spectator effects operated differently depending on team role, complicating simple narratives about crowd support.

## Health and psychology in team sport settings

The mental health and psychosocial dimensions of athlete life featured prominently across this Research Topic. A longitudinal study of 843 adolescent, Chinese footballers identified four distinct dual-career stress profiles using latent profile analysis (Quan et al.). Athletes within the most distressed profile showed the strongest association between stress and burnout and the sharpest academic decline, highlighting the need for early identification of at-risk athletes in youth development systems. Complementing this, a cross-cultural validation study confirmed that the Athlete Psychological Strain Questionnaire can be reliably used in Romanian-speaking athletes, supporting its integration into the Sport Mental Health Assessment Tool-1 screening pathway (Dragoiu et al.). Importantly, while the tool performed well for anxiety and depression, its diagnostic capacity for drug use and disordered eating was limited, warranting further investigation. In elite female basketball, a longitudinal study reported that menstrual cycle phase *per se* was not a strong predictor of sleep quality or recovery-stress states with the burden of daily symptoms being the consistent determinant (Kullik et al.). This nuanced finding challenged phase-based assumptions and called for symptom-level monitoring of female athletes. Furthermore, an investigation into narcissistic personality traits in athletes identified that body image had a low but significant influence on narcissistic admiration and rivalry, and that prolonged social media use modulated these patterns in individual-sport athletes (Kilic et al.).

Leadership and coaching effectiveness were also examined through both quantitative and conceptual perspectives within the Research Topic. A structural equation modeling study of Korean university footballers reported that transformational leadership did not directly predict team task cohesion with cognitive trust mediating the relationship entirely (Kim and Kim). Affective trust was influenced by transformational leadership and by cognitive trust, but did not itself predict cohesion. These findings suggested that coaches seeking to build cohesive, high-functioning teams should first establish rational credibility before emotional attachment develops. Finally, from a clinical perspective, a perspective article argued that systemic psychotherapy applied to the team as a unit, rather than to individuals, offered a viable framework for managing dysfunctional team dynamics, particularly in groups with players exhibiting subclinical narcissistic traits (Piepiora et al.).

## Conclusions

Taken together, these 23 studies provided a valuable and complex overview of contemporary team sport science. Three overarching themes emerged: (i) the importance of contextual factors that modulated the effects of mental fatigue, timeouts, game location, and leadership; (ii) the value of having a multidisciplinary and complex perspective, as physical, cognitive, emotional, and social dimensions of performance interacted in ways that single-domain models couldn't capture; and (iii) the inter-individual variability evidenced by dual-career stress, menstrual symptoms, and cognitive load. Future research is encouraged to translate these insights into monitoring systems and interventions that are both ecologically valid and easily applicable for practitioners working in competitive team sports.

